# *Akkermansia muciniphila*: a deworming partner independent of type 2 immunity

**DOI:** 10.1080/19490976.2024.2338947

**Published:** 2024-05-08

**Authors:** Jiaqi Wang, Xiufeng Zhao, Xianhe Li, Xuemin Jin

**Affiliations:** a State Key Laboratory for Diagnosis and Treatment of Severe Zoonotic Infectious Diseases, Key Laboratory for Zoonosis Research of the Ministry of Education, Institute of Zoonosis, College of Animal Sciences, Jilin University, Changchun, China; b State Key Laboratory for Diagnosis and Treatment of Severe Zoonotic Infectious Diseases, Key Laboratory for Zoonosis Research of Ministry of Education, Institute of Zoonosis, and College of Veterinary Medicine, Jilin University, Changchun, China; c Würzburg Institute of Systems Immunology, Max Planck Research Group at the Julius-Maximilians University of Würzburg, Würzburg, Germany; d Department of Microbiology, Immunology and Molecular Genetics, University of California Los Angeles, Los Angeles, USA

**Keywords:** *Akkermansia muciniphila*, gut microbiota, helminth, *Trichinella spiralis*, immunity

## Abstract

The gut microbiota has coevolved with the host for hundreds of millions of years, playing a beneficial role in host health. Human parasitic helminths are widespread and pose a pervasive global public health issue. Although Type 2 immunity provides partial resistance to helminth infections, the composition of the gut microbiota can change correspondingly. Therefore, it raises the question of what role the gut microbiota plays during helminth infection. *Akkermansia muciniphila* has emerged as a notable representative of beneficial microorganisms in the gut microbiota. Recent studies indicate that *A. muciniphila* is not merely associated with helminth infection but is also causally linked to infection. Here, we provide an overview of the crosstalk between *A. muciniphila* and enteric helminth infection. Our goal is to enhance our understanding of the interplay among *A. muciniphila*, helminths, and their hosts while also exploring the potential underlying mechanisms.

## 1. Introduction

Approximately 1.5 billion individuals are infected with helminth parasites, making helminths a pervasive global public health issue.^1,2^ In addition to helminths, commensal bacteria have coevolved with their hosts. The gut microbiome, comprising around 10 million bacteria, plays a critical role in maintaining the healthy metabolic status of an individual.^3,4^ The interaction between helminths and gut bacteria is crucial for determining the development of helminth infections.^5,6^

Among these commensal bacteria, *Akkermansia muciniphila* has emerged as a critical bacterium. A recent review demonstrated that the absence or reduction of *A. muciniphila* is associated with inflammatory disorders.^7^ *A. muciniphila* is vital for maintaining a healthy intestinal barrier and has been extensively studied in mice, as well as in an initial trial involving humans,^8–13^ Gaps in existing knowledge regarding the crosstalk between *A. muciniphila* and enteric helminths are being filled. In this review, we discuss the effect of enteric helminth infections on *A. muciniphila* and the role of *A. muciniphila* in enteric helminth infections. We aim to improve our understanding of *A. muciniphila*-helminth – host interactions and identify the possible underlying mechanisms.

## 2. Helminth-induced immune responses and the gut microbiota

Helminth parasites typically trigger robust type 2 immune responses that play a crucial role in eliminating these parasites.^14^ These responses become crucial as multicellular helminths migrate through host tissues.^15^ The key characteristics of helminth infections include goblet cell hyperplasia and mucin secretion in the intestines. This immune response is critical for the expulsion of helminths through the maintenance of the epithelial barrier.^16^ Goblet cells respond to type 2 cytokines by generating mucus, with well-established roles for IL-13 and IL-4.^16,17^ In the absence of IL-4 and IL-13, efficient expulsion of helminths from the mouse intestine is hindered ,^18 ,^ .^19,20^ Additionally, IL-5 deficiency in mice results in a higher worm load during both acute and chronic infections.^21,22^ These findings underscore the importance of type 2 cytokines and their signaling pathways in limiting helminth infections. Accumulating evidence suggests that the microbiota plays a pivotal role in maintaining host homeostasis, exerting significant effects on numerous disease mechanisms.^23^ Research on host – helminth interactions has focused on host-associated gut microbiomes.^24^ Notably, *Trichuris muris* larvae are unable to develop and colonize germ-free mice, underscoring the essential role of bacterial microbiota in establishing infection.^25^ The gut microbiome undergoes alterations during *T. muris* infection associated with Th2 responses.^26^ Specifically, *T. muris* infection-associated goblet cell activation restricts the colonization of *Bacteroides vulgatus*, a common member of the gut microbiota. This restriction is attributed to the activation of a robust Th2 immune response. Deworming enhances the abundance of *Bacteroidales*, suggesting a causal relationship between helminth infections and microbial diversity. *Trichinella spiralis*-induced strong type 2 immunity still occurs independently of changes in the microbiota in germ-free mice.^27^ However, fecal bacterial transplantation from healthy mice to *T. spiralis*-infected mice can increase the number of goblet cells but not the expression of IL-4 and IL-13 at day 3 post infection.^28^ Along these lines, the gut microbiota may contribute to mucus-producing goblet cell function in deworming, independent of type 2 immunity, which necessitates further investigation.

## 3. The impact of helminth infections on *A. muciniphila* abundance

The gastrointestinal tract relies on a crucial defense mechanism known as the mucus barrier. The gut microbiota, considered a vital factor influencing host health, plays a pivotal role in altering the properties of the mucus layer.^29^ Researchers at the Wageningen Laboratory of Microbiology have made groundbreaking discoveries, identifying *A. muciniphila* as a novel species within the genus *Akkermansia*, phylum *Verrucomicrobiota*, commonly inhabiting the gut mucus layer.^30^ *A. muciniphila* has been found in the human intestine since early life, without causing harm to the host.^31,32^ Remarkably, decreased abundance of *A. muciniphila* is associated with the occurrence of various diseases and physiological changes, such as obesity, diabetes mellitus, and inflammatory bowel disease,^33–35^ Researchers are particularly intrigued by the interaction between *A. muciniphila* and infectious diseases, especially helminth infections. A thorough qualitative systematic review of human studies revealed a strong association between *Akkermansia* spp. and helminth infection.^36^ For instance, individuals with gastrointestinal helminths in Sri Lanka exhibit a significant increase in *A. muciniphila* compared to uninfected individuals.^37^ Similar observations have been reported in several helminth-infected mouse models. In mice, *Heligmosomoides polygyrus* infection increases the relative abundance of *A. muciniphila*.^38,39^ Helminth-induced type 2 immunity plays a role in modulating the gut microbiota, including *A. muciniphila*.^39^ Another helminth that colonizes the intestine, *Trichuris muris*, can significantly increase the abundance of *Verrucomicrobiales*.^26^ During infection with *T. spiralis*, which inhabits the small intestine for approximately two weeks, the abundance of *Akkermansia* increases in helminth-infected mice,^40–42^ Interestingly, *Schistosoma japonicum*, which exhibits tropism for the liver, leads to an increase in *Akkermansia* abundance during *S. japonicum*-induced liver cirrhosis.^43^ Furthermore, in a mouse model of percutaneous infection with *Schistosoma mansoni*, the infected mice exhibited a greater abundance of *Akkermansia*.^44,45^

Collectively, these findings suggest that the abundance of *A. muciniphila* increases during helminth infection, potentially resulting from interactions between the helminths and their hosts. Helminth *Heligmosomoides polygyrus* infection promotes the induction of alternatively activated (M2) macrophages and helminth-induced M2 cells can increase the abundance of *A. muciniphila*. An enhanced type 2 immune response leading to increased intestinal mucus secretion is a distinctive feature of helminth infections.^46^ allows *A. muciniphila* to reach a more favorable growth environment.

## 4. The impact of *A. muciniphila* on helminth infections

**Figure 1.**
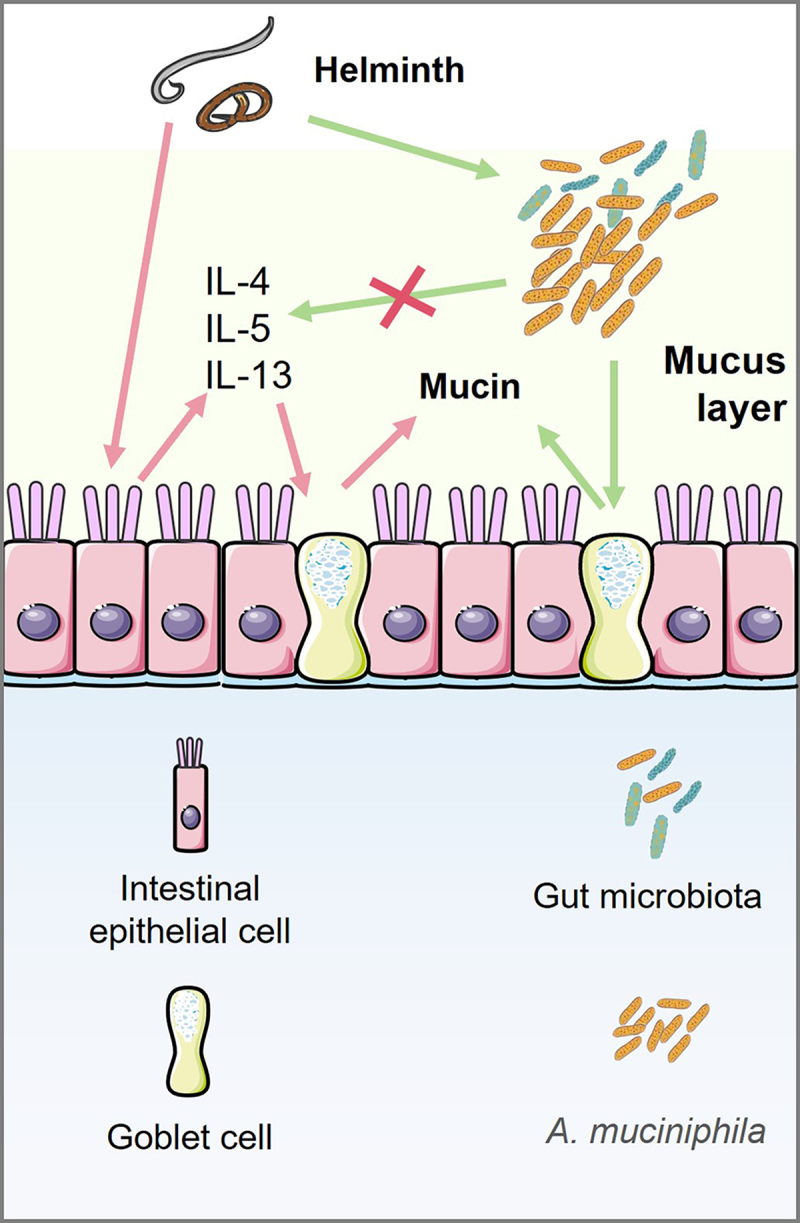
*Akkermansia muciniphila* is involved in mucin production by goblet cells independent on type 2 immunity.

A positive relationship between *A. muciniphila* colonization and helminth infection restrain has been determined (Figure 1). Our previous study demonstrated that *A. muciniphila* exerts anthelmintic effects against *T. spiralis* infection.^47^ *A. muciniphila* improved intestinal mucus secretion, and pasteurized *A. muciniphila* was more effective than live *A. muciniphila*. Another study showed that these compounds were retained even when inactivated by pasteurization.^11^ However, there is insufficient evidence to explain why pasteurized *A. muciniphila* often exhibits better efficacy. One possible explanation is that pasteurization not only has no effect on mucus-promoting bacterial components but also eliminates intestinal invasion caused by bacteria. This phenomenon has paved the way for making bacteria more suitable for various applications. To date, no studies have systematically explored the role of *A. muciniphila* in helminth infections.

Intestinal epithelial cells trigger the production of type 2 cytokines (IL-4, IL-5, and IL-13) in response to helminths. Mucus production by goblet cells is induced by type 2 cytokines. Type 2 immunity limits helminth infections and can result in their physical expulsion from the mucosal membranes where the helminth resides. In addition, the gut microbiota may contribute to mucus-producing goblet cell function during deworming. Although *A. muciniphila* cannot affect type 2 cytokines (IL-4, IL-5 and IL-13) production, *A. muciniphila* can improve intestinal mucin production, indicating the presence of an alternative type 2 immunity-independent mechanism for the mucin-promoting function of *A. muciniphila*. However, the mechanisms have not yet been elucidated.

**Figure 2.**
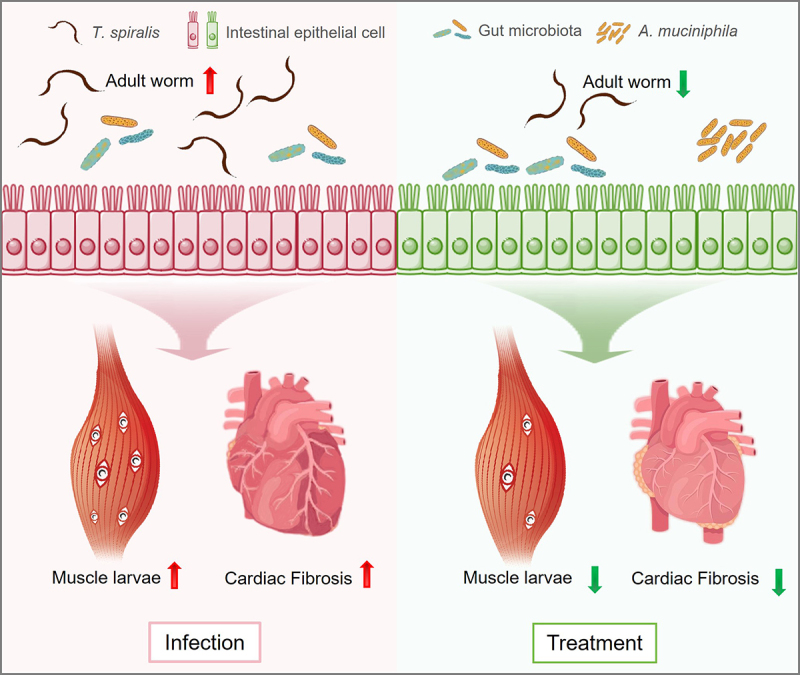
*Akkermansia muciniphila* plays a role in protection against *Trichinella spiralis* infection.

Furthermore, infection with helminth *T. spiralis* causes cardiac fibrosis (Figure 2). Recent metagenomics analysis revealed a higher presence of *A. muciniphila* in the small intestine of infected mice. Interestingly, antibiotic treatment exacerbated helminth-induced cardiac fibrosis, and the dominant species, *A. muciniphila*, during *T. spiralis* infection appeared to positively influence cardiac fibrosis outcomes.^40^ *Schistosoma* is also one of the major pathogens that causes fibrosis in the liver.^48,49^ As mentioned in above section, the expansion of *A. muciniphila* in humans and mice is induced by infection with different genera of *Schistosoma*, such as *S. mansoni* and *S. japonicum*,^43–45^ Treatment with *A. muciniphila* protects against liver fibrosis induced by a high-fat diet and carbon tetrachloride administration.^50^ *A. muciniphila* has shown potential for ameliorating liver inflammation and fibrosis in NEMO^∆hepa^ mice prone to liver carcinogenesis.^51^ However, the role of *A. muciniphila* in the development of *Schistosoma*-induced liver fibrosis has not been determined. Additionally, it has been observed that compounds present in the outer membrane of *A. muciniphila* (Amuc_1100) mechanically bind to the toll-like receptor (TLR) 2.^11^ Our previous studies have confirmed the involvement of TLR2 in the effects of pasteurized *A. muciniphila* on helminth infection.^40,47^ We conducted an experiment involving TLR2 knockout mice and found that treatment with pasteurized *A. muciniphila* failed to alleviate the helminth burden and pathology associated with *T. spiralis* infection.^40,47^

In the left panel, infection with *T. spiralis* leads to helminth burdens, including adult worms and muscle larvae, and causes cardiac fibrosis. As shown in the right panel, treatment with *A. muciniphila* reduced the burden on adult worms and muscle larvae and ameliorated the severity of cardiac fibrosis induced by *T. spiralis*.

While *A. muciniphila* has a deworming effect, it does not enhance the type 2 immune response, as evidenced by no increase in IL-4 concentration.^52^ No evidence supports a correlation between *A. muciniphila* and other type 2 cytokines. Although *A. muciniphila* cannot induce Th2 cell response,^53^ indicating the presence of an alternative type 2 immunity-independent mechanism for the mucin-promoting function of *A. muciniphila*, more thorough mechanistic studies using a type 2 immunity-deficient helminth-infected model should be conducted in future. A recent study indicated that 15:0-i15:0 phosphatidylethanolamine, a lipid found in the cell membrane of *A. muciniphila*, increases the release of tumor necrosis factor (TNF)-α in a TLR2-dependent manner.^54^ Moreover, TNF-α plays a crucial role in the expulsion of *T. spiralis*,^55^ suggesting its putative involvement in the capacity of *A. muciniphila* to expel *T. spiralis*. *A. muciniphila* promotes the differentiation of secretory intestinal epithelial cell lineages, accelerates intestinal epithelial regeneration and increases the number of mucin-producing goblet cells in the intestines, consequently enhancing mucus production during gut damage caused by radiation and methotrexate.^56^ Supplementation with *A. muciniphila* prevents the decrease in thickness of the colonic mucus layer that occurs with aging.^57^ *A. muciniphila* promotes the development of goblet cells in the intestine, resulting in increased mucin production^58^ and enhancing the integrity of the gut barrier.^8^ *A. muciniphila* also upregulates the genes responsible for maintaining intestinal barrier function (e.g., MUC2, BIRC3, and TNFAIP3) and improves intestinal homeostasis by activating the ALPK1/TIFA/TRAF6 axis.^59^ Furthermore, *A. muciniphila* stimulates NLRP6 expression and enhances autophagy in goblet cells, thereby promoting the production of mucin in the context of inflammatory bowel disease.^58^ These findings provide mechanistic and novel insights into *A. muciniphila*-induced mucus production. However, the mechanisms underlying helminth infection have not yet been fully elucidated.

## 5 Conclusions

*A. muciniphila*-induced regulation of gut barrier function, including mucus production, has been identified by various research teams. Although the relevance of *A. muciniphila* in helminth infections has been described in many publications, the studies about relationship between *A. muciniphila* colonization and helminth infection restrain have just begun. Indeed, although some studies have argued that mucin-degrading bacteria have a risk of reducing the mucus layer due to their capacity for mucus consumption,^60,61^ improved mucus production by *A. muciniphila* during helminth *T. spiralis* infection is crucial for its anthelmintic effects. Given that increasing goblet cell mucus secretion constitutes part of the “weep and sweep” response that develops to promote helminth expulsion,^14^ helminth infection could be considered a model for investigating the crosstalk between *A. muciniphila* and mucus. The protective role of *A. muciniphila* and its associated molecules against infection with other helminths should be explored further. Moreover, the detailed mechanism of the type 2 immune independent mechanism by which *A. muciniphila* regulates mucin function in helminth infections is worth exploring in the future.

## Data Availability

All data are included in the manuscript.
